# The Roles of Beclin 1 Expression in Gastric Cancer: A Marker for Carcinogenesis, Aggressive Behaviors and Favorable Prognosis, and a Target of Gene Therapy

**DOI:** 10.3389/fonc.2020.613679

**Published:** 2020-12-23

**Authors:** Hua-chuan Zheng, Shuang Zhao, Hang Xue, En-hong Zhao, Hua-mao Jiang, Chang-lai Hao

**Affiliations:** ^1^ Department of Oncology and Experimental Center, The Affiliated Hospital of Chengde Medical University, Chengde, China; ^2^ Department of Surgery, The Affiliated Hospital of Chengde Medical University, Chengde, China; ^3^ Department of Urology, The First Affiliated Hospital of Jinzhou Medical University, Jinzhou, China; ^4^ Department of Hematology, The Affiliated Hospital of Chengde Medical University, Chengde, China

**Keywords:** Beclin 1, gastric cancer, gene therapy, carcinogenesis, aggressive behaviors, prognosis

## Abstract

Beclin 1 is encoded by *Becn1*, and plays a role in tumorigenesis, neurodegeneration, apoptosis and autophagy. Here, the aggressive phenotypes and relevant proteins were examined after Beclin 1 expression was altered in gastric cancer cells. We also observed the effects of Beclin 1 on gastric carcinogenesis using *Becn1* knockout mice. Finally, clinicopathological significances of Beclin 1 expression were analyzed using meta- and bioinformatics analyses. *Becn1* overexpression was found to inhibit proliferation, glucose metabolism, migration and invasion of gastric cancer cells, whereas its knockdown caused the opposite effects. Beclin 1 suppressed the tumor growth by decreasing proliferation and increasing apoptosis. The heterozygous abrogation of *B*e*cn1* in gastric pit, parietal and chief cells could not cause any epithelial lesion. Beclin 1-mediated chemoresistance was closely linked to the autophagy, Bax underexpression, and the overexpression of Bcl-2, LRP1, MDR1, and ING5. Bioinformatics analysis showed higher Becn1 mRNA expression in intestinal- than diffuse-type carcinomas (*P*<0.05), and in male than female gastric cancer patients (*P*<0.05). *Becn1* hyperexpression was positively associated with both overall and progression-free survival rates of the cancer patients (*P*<0.05). Meta-analysis showed that down-regulated Beclin 1 expression in gastric cancer was positively with lymph node metastasis, TNM staging, dedifferentiation and poor prognosis (*P*<0.05). *Becn1*-related signal pathways in gastric cancer included prostate, lung, renal, colorectal, endometrial and thyroid cancers, glioma, and leukemia, the metabolism of amino acid, lipid and sugar, and some signal pathways of insulin, MAPK, TRL, VEGF, JAK-STAT, chemokine, p53, lysosome, peroxidome and ubiquitin-mediated protein degradation (*P*<0.05). These suggested that Beclin 1 might be considered as a potential marker of gastric carcinogenesis, aggressiveness and prognostic prediction, and as a target of gene therapy in gastric cancer.

## Introduction

There are a worldwide decrease in morbidity and mortality and rapid development in diagnostic and operative techniques, but gastric cancer still challenges the human health. The clinical outcome and prognosis of gastric cancer are generally worse because it has often metastasized by the time of discovery and most cancer patients are elderly at presentation (1, 2). Therefore, deep clarification of molecular mechanisms about gastric carcinogenesis and subsequent progression may remarkably improve early finding, diagnosis and treatment.

Autophagy is a conserved intracellular degradation pathway *via* autophagosomes formation, essential for protein development, homeostasis and survival, and mainly mediated by Beclin 1 (3, 4). EHMT2/G9a hypoexpression reduced H3K9me2 level, and dissociation of EHMT2 with H3K9me2 from *Becn1* promoter, and epigenetically up-regulated *Becn1* transcription *via* a high ROS level and NF-κB activation (5). Additionally, Beclin 1 binds to Bcl-2, Bcl-w, and Bcl-xL *via* BH3 receptor domain by phosphorylation and ubiquitination of Beclin 1 (6). Beclin 1 is phosphorylated at ser-90 by PP2A and DAPK3 to control autophagy (7). The phosphorylated Beclin 1 at S409 by CK1 is essential for p300 binding and Beclin 1 acetylation at lysine 430 and 437 (8). RNF216 interacts with and ubiquitinates Beclin 1 at lysine 48, thereby contributing to Beclin 1 degradation, while SLC9A3R1 and USP19 blocks ubiquitin-dependent Beclin 1 degradation by interacting with Beclin 1 and removing the K11-linked ubiquitin respectively (9, 10). Liu et al. (11) found that Beclin 1, the core molecule of the Becn1-PIK3C3 complex, could be SUMO3-conjugated by PIAS3 predominantly at K380 and deSUMOylated by SENP3. Tang et al. (12) reported that 14-3-3ζ bound to and stabilized phospho- Beclin 1(S295), and induced autophagy and chemoresistance in hepatocellular carcinoma cells. Caspase 3-mediated hydrolysis of Beclin 1 suppresses autophagy or enhances apoptosis by translocating Bax to the mitochondria for the release of cytochrome c (13, 14), whereas ABHD5 directly competes with Caspase 3 for binding to Beclin 1, and consequently prevents Beclin 1 from cleavage (15).

Previously, Beclin 1 overexpression in ovarian cancer was reported to negatively correlate with the differentiation and higher cumulative and relapse-free survival rates (16). Either Beclin 1 mRNA or protein hyperexpression was seen in colorectal and gastric cancers in comparison to precancerous lesion or mucosa. Beclin 1 expression was inversely associated with liver metastasis and distant metastasis of colorectal cancer, or venous invasion, lymph node metastasis, TNM staging, dedifferentiation and favorable prognosis of gastric cancer (17, 18). In colorectal cancer cells, Beclin 1 inhibited cell viability, migration and invasion, lamellipodia formation, and tumor growth, induced autophagy and apoptosis (19). Reportedly, Beclin 1 overexpression augmented the apoptotic induction of cis-diamminedichloroplatinum *via* enhancing Caspase-9 activity in gastric cancer cells (18). Gao et al. (20) reported that radiotherapy also induced autophagy and increased Beclin-1 expression *via* p53 pathway. Biallelic loss of *Becn1* is embryonically lethal for knockout mice, and promotes spontaneous tumorigenesis of lymphomas, liver and lung cancers (21–24). Here, we analyzed the effects of Beclin 1 expression on the aggressive behaviors and phenotypes of gastric cancer cells, and clarified relevant mechanisms. To explore the role of *Becn1* knockout in gastric carcinogenesis, we established conditional *Becn1* knockout mice in gastric pit, parietal and chief cells using the Capn8, Atp4b and PGC promoter to initiate Cre recombination respectively. Finally, clinicopathological significances were analyzed using meta- and bioinformatics analyses.

## Materials and Methods

### Cell Line and Culture

Gastric cancer cell lines (BGC-823 and MKN28) were kindly presented by Prof. Su of Jinzhou Medical University. These cells were grown in RPMI 1640 medium containing FBS, penicillin, and streptomycin in 5% CO_2_ at 37°C. BGC-823 and MKN28 cells were subjected to pcDNA3.1-*Becn1* or pcDNA3.1 vector transfection, and selected by G418 with monoclone collection. The target sequences of sh-*Becn1* were 5’-CACCGGAATGGAATGAGATTAATGCTTCAAGAGAGCATTAATCTCATT CCATTCCTTTTTTG-3’ and 3’-CCTTACTTACTCTAATTACGAAGTTCTCTCGTAATTAGAGTAA GGTAAGGAAAAAACCTAG-5’. Additionally, we treated cells with MG132 (proteasome inhibitor), paclitaxel (mitotic inhibitor), or SAHA (histone deacetylase inhibitor).

### Proliferation Assay

We used cell counting kit-8 (CCK-8) to determine cell proliferation. In brief, 2.0 × 10^3^ cells/well were cultured on 96-well plate. After adhering to plate.10 μl of CCK-8 solution was added into each well of the plate at different time points, and the absorbance was measured at 450 nm after 3 h incubation.

### Cell Cycle Analysis

We trypsinized, collected, and fixed the cell using ethanol for 2 h. After RNase treatment for 1 h, cells were pelleted and stained by propidium iodide (PI) for 30 min. At final, flow cytometry was used to examine PI signal.

### Apoptosis Assay

FITC-labeled annexin V staining (Kagen, China) was employed to indicate phosphatidylserine externalization of early apoptosis in terms of manufacturer’s instructions.

### Measurement of Extracellular Acidification Rate and Oxygen Consumption Rate

We measured the metabolic parameters using wing discs of abxUbxFLPase of Seahorse metabolic flux analyzer. Discs were measured in bicarbonate-free Schneider medium with 12 mM glutamine and 11 mM glucose. The glycolysis can be indicated by extracellular acidification rate (ECAR), and the mitochrondrial respiration by oxygen consumption rate (OCR). We also normalized ECAR and OCR values using protein amount according to BCA assay.

### Transwell Chamber Assay

To assess invasion, we cultured 2.0 × 10^5^ cells in FBS-free medium in the matrigel-coated insert on the chamber top, and added 10%-FBS-containing medium to the chamber bottom as a chemoattractant. After 24-h incubation, the upper member of insert was scrubbed, and the lower portion fixed in methanol and followed by Giemsa dye. To measure migration, we repeated the above-mentioned protocol excluding membrane-control insert.

### Animals

We housed three mice per plastic cage with paper chips, standard rodent food and water in pathogen-free and temperature-controlled condition with a 12-h light/dark illumination cycle. All animal experiments were conducted using protocols approved by the Committee on Animal Experimentation of The Affiliated Hospital of Chengde Medical University. Female 8-week Balb/c nude mice were used for subcutaneous implantation by injection of 2.0× 10^6^ cells per mouse to axilla (n=10 mice/group). Finally, the mice were anesthetized, photographed, and killed for tissue sampling. The part of tumors was subjected to routine pathological block preparation, and the remaining was stored in liquid nitrogen until protein, RNA or protein extract. The tumor volume was calculated as width^2^× length ×0.52.

Additionally, we performed cre-mediated deletion of floxed alleles by mating *Becn1* conditional mutant mice (Mouse Biology Program, University of California, Davis USA) with Capn8-cre, Atp4b-cre (kindly presented by Prof. Yang) or PGC-cre (prepared in our Lab., unpublished) transgenic mice. At least 5 mice were killed at 9 month, and their stomachs were analyzed.

### DNA Analysis

DNA was extracted from mouse tail and stomach using phenol-chloroform method. We performed genotyping by PCR. The PCR primer sequences were CSD-lacF: 5’-CTACCATTACCAGTTGGTCT GGTGTC-3’, CSD-neoF: 5’-GGGATCTCATGCTGGAGTTCTTCG-3’, CSD-*Becn1* -F: 5’-TTGTAC CGTGATTTAGGGCGTTTGC-3’, CSD-*Becn1*-R: 5’-CAGAGTGAGTTCCAAGACAGCCAGG-3’, CSD-*Becn1*-ttR: 5’-CTCCCAAGTGCTGGGATTAAAGACG-3’ and cre: 5’-GCCTGCATTACCGG TCGATGC-3’ and 5’-CAGGGTGTTATAAGCAATCCC-3’. The truncated *Becn1* and cre were confirmed by the tail DNA PCR. The deletion of *Becn1* was confirmed by PCR amplification of gastric mucosa DNA.

### Real-Time RT-PCR

We extracted total RNA from cells or tissues using Trizol (Takara). Reverse transcription of 1 µg RNA was performed using random primers and AMV reverse transcriptase. PCR primers were designed according to the sequences in GenBank. Oligonucleotide primers for PCR were 5’-TTACCACAGC CCAGGCGA-3’ and 5’-GCCACCATCAGATGCCTC-3’ for mouse *Becn1*, and 5’-ACATACTCAG CACCGGCCTC-3’ and 5’-TATGACTCCACTCACGGCAAA-3’ for mouse *GAPDH*. SYBR Premix Ex Taq II kit (Takara) was employed to amplify target cDNAs using *GAPDH* as an internal control.

### Western Blot

Protein was extracted in RIPA lysis buffer and determined by BCA assay. We separated denatured protein in SDS-polyacrylamide gel and transferred to Hybond membrane. After blocked in 5% skim milk, the membranes were incubated with primary antibodies (**Table 1**) and then with anti-rabbit, or anti-mouse IgG conjugated to horseradish peroxidase (Dako). We visualized bands using ECL-Plus detection reagents.

**Table 1:** The antibodies used in the present study.

Name	Source	Company
Beclin 1	Rabbit	Santa Cruz
β-actin	Mouse	Santa Cruz
GAPDH	Rabbit	Wanleibio
LC3B	Rabbit	Wanleibio
Bax (B-9)	Mouse	Santa Cruz
Bcl-2 (C-21)	Rabbit	Santa Cruz
Akt	Rabbit	Santa Cruz
PI3K	Rabbit	Abcam
p-p38	Rabbit	Santa Cruz
P38	Rabbit	Santa Cruz
NF-ĸB	Rabbit	CST
Cdk4(C-22)	Rabbit	Santa Cruz
E-cadherin	Mouse	Abcam
Twist1	Rabbit	Abcam
Zeb2(E-11)	Mouse	Santa Cruz
β-catenin	Rabbit	Abcam
MRP1	Mouse	Abcam
GSTπ	Rabbit	Abcam
MDR1	Rabbit	Abcam
FBXW7	Rabbit	Wanleibio
LRP1	Rabbit	Abcam
ING5	Rabbit	Proteintech
CD147	Rabbit	Abcam

### Histological Analysis

Tissues block were sectioned and consecutive 5μm-thick sections were stained with HE, alcian blue, PAS and HID. Sections were deparaffinized, dehydrated, and subjected to immunostaining using primary antibodies (**Table 1**) as previously described (17), or TUNEL using ApopTag Plus Peroxidase In Situ Apoptosis Detection Kit (Millipore) as reported (19).

### Identification of Eligible Studies

We searched the articles using BIOSIS, PubMed, SciFinder, and Web of Science until July 10, 2020. The following strategy was (*Becn1* OR Beclin 1) AND (stomach OR gastric) AND (adenocarcinoma OR carcinoma OR cancer). The articles were included to observe Beclin 1 immunostaining in gastric cancers and compare Beclin 1 expression with their pathobiological features and prognosis. We excluded the abstract, comment, review and meeting, and papers about Western blot, RT-PCR, cDNA chip, or transcriptomic data for Beclin 1 expression.

### Data Extraction and Quality Score Assessment

Both reviewers independently collected useful information from all eligible articles, including first author, publication year, ethnicity, country, histological subtypes, control source, antibody company, numbers of cases and controls, and survival times. Engauge Digitizer software was employed to mine information from Kaplan-Meier curves and calculate Hazard ratios. We discussed to resolve disagreement until consensus. Publication quality was evaluated according to Newcastle Ottawa Scale.

### Bioinformatics Analysis

The transcriptomic and clinicopathological data of gastric cancer patients were downloaded from TCGA database by TCGA-assembler of R software. We analyzed Becn1 mRNA level between gastric normal and cancer tissues using Oncomine and TACGA data. *Becn1* expression was compared with clinicopathological and prognostic data of gastric cancer patients. GSEA was performed with GSEA-3.0. We used *Becn1* level as a phenotype label and analyzed pathway enrichment. Kaplan- Meier plotter was employed to analyze the prognostic significance of Becn1 mRNA.

### Statistics Analysis

We used Revman software 5.3 to carry out Meta-analysis, and evaluated HWE using Chi-square test. If there was no significant heterogeneity, the fixed effect model would be employed. Experimental and TCGA data was handled with SPSS 10.0 software using t test or survival analysis. We regarded *P*<0.05 as statistically significant.

## Results

### The Effects of Beclin 1 Overexpression on the Aggressiveness of Gastric Cancer Cells

**Figure 1:**
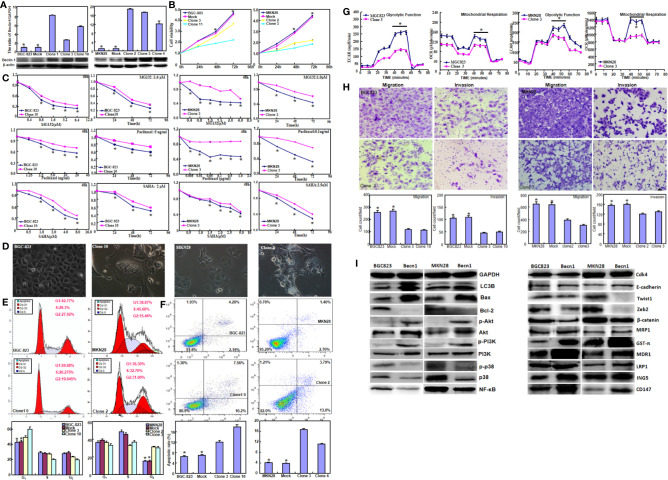
The effects of Beclin 1 expression on aggressiveness of gastric cancer cells. Beclin 1 expression in BGC-823 and MKN28 cells was examined after transfection with pcDNA3.1-*Becn1* by real-time RT-PCR and Western blot **(A)**. The transfectants showed a decrease in growth **(B)** and chemosensitivity to MG132, Paclitaxel and SAHA **(C)** in comparison with the control or mock. After Becn1 was overexpressed, the morphological appearance was observed under microscope **(D)**. Flow cytometry showed that Beclin 1 expression induced the G_1_ arrest of BGC-823 cells, but S arrest of MKN28 cells **(E)**. Apoptosis was increased by Beclin 1 overexpression, as evidenced by Annexin V assay **(F)**. Oxygen consumption and transwell assays were used to monitor the glucose metabolism, migration and invasion of gastric cancer cells respectively **(G, H)**. The phenotype-related proteins were screened by Western blot **(I)**. *P < 0.05, compared with the transfectants.

Beclin 1 was successfully overexpressed in BGC-823 and MKN28 cells according to real-time RT-PCR and Western blot (**Figure 1A**, *P*<0.05). Beclin 1 overexpression suppressed the proliferation of both cancer cells, evidenced by CCK-8 (**Figure 1B**, *P*<0.05). *Becn1* transfectants showed the chemoresistance to MG132, paclitaxel and SAHA at both time- and dose-dependent manners (**Figure 1C**, *P*<0.05). After transfected with *Becn1*-expressing plasmid, the appearance of gastric cancer cells became irregular, vacuolar, polynucleate and protrudent (**Figure 1D**). Flow cytometry showed G_1_ or S arrest, and apoptotic induction in *Becn1* transfectants of gastric cancer cells (**Figures 1E, F**, *P*<0.05). Beclin 1 overexpression decreased glycolysis and following mitochondrial oxidization, cell migration and invasion according to oxygen consumption and transwell assays respectively (**Figures 1G, H**, *P*<0.05). As shown in **Figure 1I**, *Becn1* overexpression up-regulated the expression of LC-3B, Bax, Akt, p-Akt, PI3K, p-PI3K, E-cadherin, GST-π, MDR1, LRP1, ING5 and CD147, but down-regulated the expression of Bcl-2, p38, p-p38, NF-κB, Twist, Zeb2 and β-catenin. There was no difference in the expression of Cdk4 and MRP1 between gastric cancer cells and their *Becn1* transfectants.

**Figure 2:**
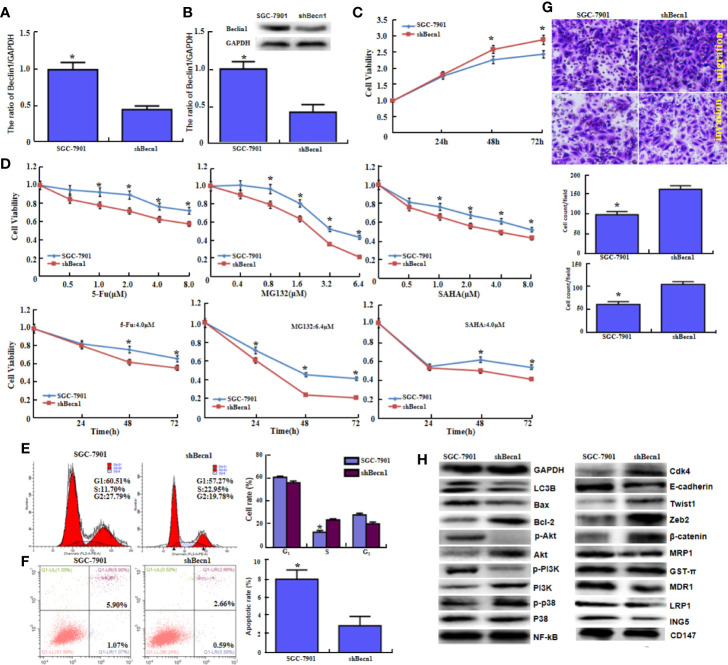
The *in vitro* and *in vivo* effects of Beclin 1 silencing on aggressive phenotypes of gastric cancer cells. After transfection of shRNA-*Becn1*, its expression became weak in SGC-7901 cells by real-time RT-PCR **(A)** and Western blot **(B)**. The cell viability was measured in SGC-7901 cells and their sh*Becn1* transfectants using CCK-8 kit **(C)**, even treated with 5-FU, MG132, and SAHA **(D)**. Cell cycle and apoptosis were examined by propidium iodide (PI) **(E)** and Annexin-V **(F)** staining respectively. Both migration and invasion were determined in SGC-7901 and sh*Becn1* transfectants by transwell chamber assay **(G)**. The phenotype-associated proteins were screened by Western lot **(H)**. *P < 0.05, compared with the transfectants.

*Becn1* was successfully silenced in SGC-7901 cells, evidenced by real-time RT-PCR (**Figure 2A**, *P*<0.05) and Western blot (**Figure 2B**, *P*<0.05). *Becn1* knockdown promoted the cell viability (**Figure 2C**, *P*<0.05) and the chemosensitivity against MG132, 5-FU, and SAHA (**Figure 2D**, *P*<0.05), induced S phase progression (**Figure 2E**, *P*<0.05), and inhibited the apoptosis (**Figure 2F**, *P*<0.05). Down-regulated *Becn1* expression promoted migration and invasion according to transwell chamber assay (**Figure 2G**). *Becn1* knockdown decreased the expression of p-Akt, p-PI3K, LC-3B, E-cadherin, MRP1, MDR1, LRP1 and ING5, but increased the expression of Bcl-2, Akt, PI3K, p-p38, Cdk4, Zeb2, and β-catenin. No alteration was observed in the expression of GST-π, NF-κB and CD147 (**Figure 2H**).

**Figure 3:**
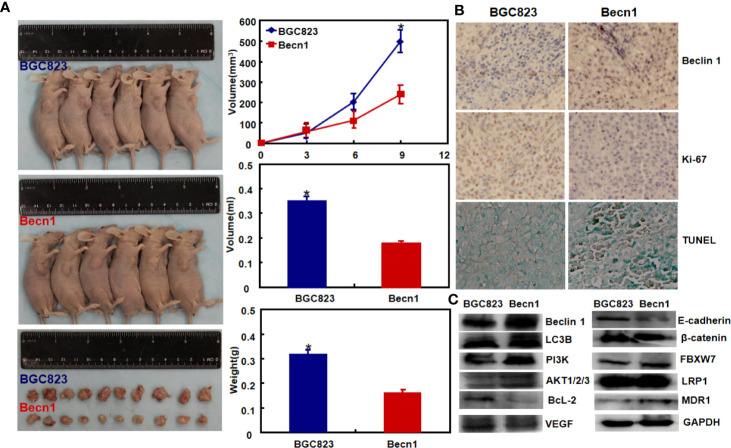
The effects of *Becn1* expression on tumor growth of gastric cancer. The growth of gastric cancer cells was faster and heavier than their *Becn1* transfectants by gross appearance, and measuring tumor volume and weight [**(A)**, *P* < 0.05]. The transfectant cells showed stronger signals of Beclin 1 and apoptosis than the control in subcutaneous tumor, but versa for ki-67 immunoreactivity **(B)**. The phenotype-related proteins were screened using xenograft tumor tissues by Western blot **(C)**. *P < 0.05, compared with the transfectant.

**Figure 4:**
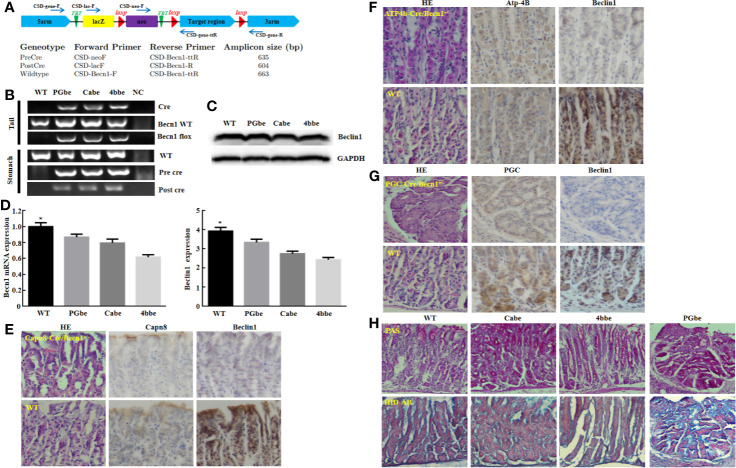
The effects of *Becn1* expression on gastric carcinogenesis. PCR primers were designed **(A)** and subjected to PCR of tail and stomach DNA **(B)**. Monoallelic deletion of *Becn1* was confirmed by real-time PCR **(C)** and Western blot **(D)**, and couldn’t cause any gastric lesions although Beclin 1 expression was reduced with Capn8, Atp-4B, and PGC immunoreactivity as control according to HE and immunohistochemistry **(E–G)**. There was no difference in alcian blue, PAS and HID staining between three conditional knock-out mice and wild-type mice **(H)**. *P < 0.05, compared with the knockout mice. WT, wild-type mice; Cabe, Capn8-cre +; *Becn1* +/−; 4bbe, Atp-4b-cre +; *Becn1* +/−; PGbe, PGC-cre +; *Becn1* +/−.

Moreover, Beclin 1 overexpression suppressed tumor growth of gastric cancer cells in nude mice (**Figure 3A**, *P*<0.05). There was a higher Beclin 1 expression, a stronger signal of TUNEL and a weaker ki-67 expression in tumors of *Becn1*-overexpressing BCG-823 (**Figures 3B, C**). Beclin 1 down-regulated the expression of VEGF, E-cadherin, Bcl-2, but up-regulated the expression of LC-3B, NF-κB, PI3K, Akt1/2/3 and MDR in xenograft tumor of gastric cancer cells (**Figure 3C**). Moreover, we matched the conditional *Becn1*-knockout (KO) mice with Atp4b-cre, Capn8-cre or PGC-cre mice, and designed the primers to confirm the monoallelic deletion of *Becn1* using DNA from tail and gastric mucosa (**Figure 4A**). According to PCR results, we obtained the monoallelic deletion of *Becn1* in gastric mucosa in *Becn1*-PGC-cre, *Becn1*-Capn8-cre and *Becn1*-Atp4b-cre mice (**Figure 4B**). There was a weaker expression of *Becn1* in gastric epithelium of *Becn1*-PGC-cre, *Becn1*-Capn8-cre and *Becn1*-Atp4b-cre than those of wild-type (WT) mice at both mRNA and protein levels (**Figure 4C, D**). However, no remarkable lesions were observed in the gastric mucosa of these three kinds of mice although weaker Beclin 1 was immunohistochemically observed in gastric chief, pit and parietal cells of *Becn1*-PGC-cre, *Becn1*-Capn8-cre, and *Becn1*-Atp4b-cre than those of WT mice (**Figures 4E–G**). Additionally, there were no differences in sialic acid, neutral and sulfuric acid mucus between conditional KO and WT mice according to alcian blue, PAS and HID staining (**Figure 4H**).

### Characteristics of Eligible Studies

We retrieved fifteen articles about the relationship between Beclin 1 expression and cancer risk, clinicopathological or prognostic features of gastric cancer for meta-analysis (**Table 2**). Five pieces of paper had the samples of normal gastric mucosa and cancer (18, 28, 31, 35, 37). In 12 articles, Beclin1 expression was compared with age, invasive depth, lymph node involvement, TNM staging and Lauren’s classification (18, 25–37). Ten pieces of paper described the prognostic significance of Beclin 1 expression (18, 25–27, 30–34, 36).

**Table 2:** Main characteristics of eligible studies.

First author	Year	Country	Ethnicity	Antibody Source	Cases	Ctr	Risk	Outcome	Quality
Won KY (25)	2015	Korea	Asian	Abcam	108		Down	Pos	8
Yu M (18)	2013	China	Asian	Sigma	565	586		Pos	9
Geng QR (26)	2012	China	Asian	Novus Biologicals	271		Up	Pos	8
Chen YB (27)	2012	China	Asian	Abcam	155	60	Up	Pos	9
Fei BY (28)	2016	China	Asian	Abcam	75	75			9
Qin WJ (29)	2015	China	Asian	Abcam	156				8
Zhou WH (30)	2012	China	Asian	Santa Cruz	153		Down	Pos	8
Yu SJ (31)	2016	China	Asian	Sigma	160	34	Down	Pos	9
Zhao Z (32)	2015	China	Asian	Abcam	113		Up	Neg	8
Qiu GL (33)	2016	China	Asian	Abcam	96	96	Up	Neg	9
Cao QH (34)	2016	China	Asian	Cell Signaling	352		Down	Pos	8
Huang JL (35)	2016	China	Asian	CST	120	120	Down		9
Hu YF (36)	2017	China	Asian	abcam	120	120	Up	Pos	9
Guo CQ (37)	2010	China	Asian	Santa cruz	62	36	Down		8

Crt, control; Down, down-regulated; Up, up-regulated; Pos, positive, Neg, negative.

### Association Between Beclin 1 Expression and Clinicopathological Parameters of Gastric Cancer

**Figure 5:**
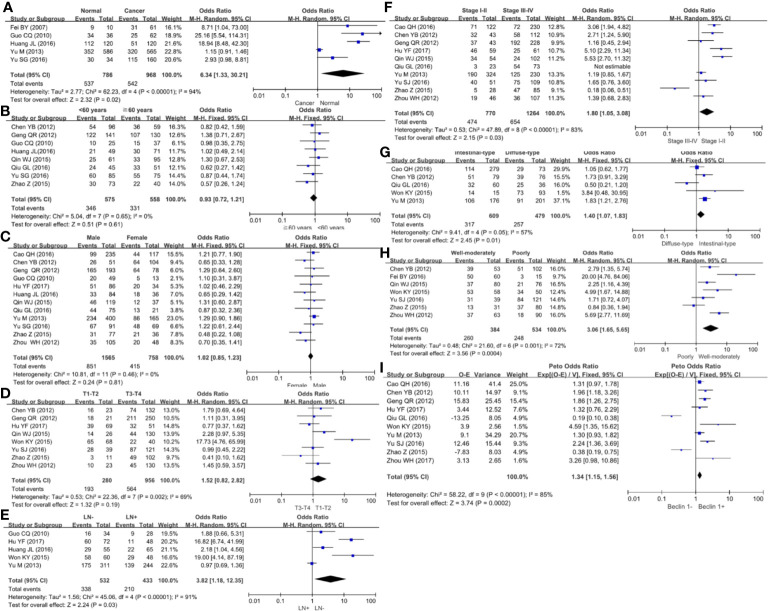
Forest plot for the relationship between Beclin 1 expression and clinicopathological parameters of gastric cancer. **(A)** gastric carcinogenesis (cancer *vs* normal mucosa); **(B)** correlation between age and Beclin 1 expression (<60 years *vs* ≧60years); **(C)** correlation between gender and Beclin 1 expression (female *vs* male); **(D)** correlation between T staging and Beclin 1 expression (Tis-1 *vs* T2-4); **(E)** correlation between lymph node metastasis (LN) and Beclin 1 expression (LN- *vs* LN+); **(F)** correlation between TNM staging and Beclin 1 expression (stage I–II *vs* II–IV); **(G)** correlation between Lauren’s classification and Beclin 1 expression (intestinal-type *vs* diffuse-type); **(H)** correlation between WHO’ classification and Beclin 1 expression (Well-moderately differentiated *vs* poorly-differentiated); **(I)** correlation between prognosis and Beclin 1 expression (Beclin 1- *vs* Beclin 1+).

We collected 986 cancers and 786 controls and found down-regulated Beclin 1 expression in gastric cancer (**Figure 5A**, *P*=0.02). As shown in **Figure 5B**, there was no difference in Beclin 1 expression between age >60 year and <60 year cancer patients (*P*>0.05), between male and female ones (**Figure 5C**, *P*>0.05) or between T_1-2_ than T_3-4_ gastric cancer (**Figure 5D**, *P*=0.19). Beclin 1 expression was negatively associated with lymph node involvement (**Figure 5E**, *P*=0.03), TNM staging (**Figure 5F**, *P*=0.03), and dedifferentiation (**Figure 5G**, *P*=0.01). Well-moderately adenocarcinoma had a higher Beclin 1 expression than the poorly-differentiated subtype (**Figure 5H**, *P*=0.0004). As indicated in **Figure 5I**, there was significant association between Beclin 1 expression and favorable overall survival in patients with gastric cancer (OR=1.34, 95% CI: 1.15–1.56, *P*=0.0002). Sensitivity analysis was performed to evaluate individual study’s influence on the pooled results by deleting one single study (data not shown).

### The Clinicopathological and Prognostic Significances of Becn1 Expression in Gastric Cancer

**Figure 6:**
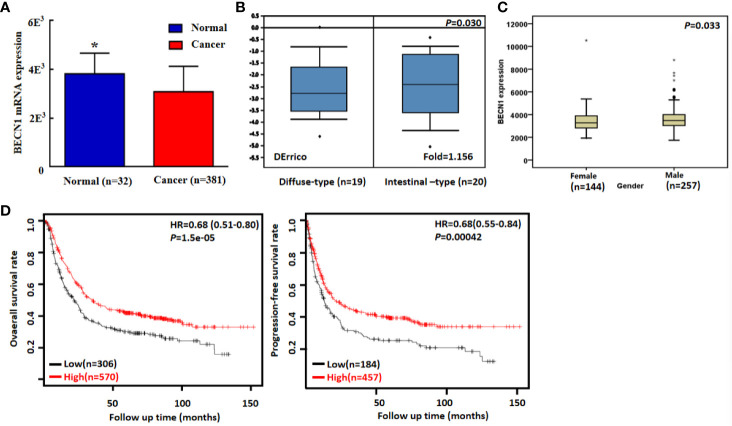
*Becn1* mRNA expression in gastric carcinogenesis and subsequent progression. A lower *Becn1* mRNA expression was detectable in gastric cancer than that in normal gastric mucosa according TCGA data [**(A)**, *P* < 0.05]. There was *Becn1* overexpression in intestinal-type than diffuse-type carcinoma according to DErrico’s database [**(B)**, *P* < 0.05]. TCGA database showed a higher *Becn1* mRNA expression in male than female patients with gastric cancer [**(C)**, *P* < 0.05]. According to the data from Kaplan-Meier plotter, *Becn1* mRNA expression was positively related to both overall and progression-free survival rates of the patients with gastric cancer [**(D)**, *P* < 0.05]. HR, hazard ratio. *P < 0.05, compared with the cancer.

According to TCGA databases, Becn1 mRNA expression was found to be lower in gastric cancer than normal mucosa (**Figure 6A**, *P*<0.05). There was a higher Becn1 mRNA expression in intestinal- than diffuse-type adenocarcinoma according to DErricodata (**Figure 6B**, *P*<0.05). In TCGA data, Becn1 expression was higher in male than female cancer patients (**Figure 6C**, *P*<0.05). According to Kaplan-Meier plotter, Becn1 expression was found to positively correlate with overall and progression-free survival rates of all cancer patients (**Figure 6D**, *P*<0.05), even stratified by Lauren’s classification, TNM staging and treatment (**Table 3**, *P*<0.05). Female, male, T2, T3, N1-N3, M1, M0 and Her-2-negative cancer patients with high Becn1 mRNA expression had a high overall survival rate than those with its low expression (*P*<0.05), while it was the same for progression-free survival rate in the male, T2, T4, N1-3, N1, M0, Her2-negative and -positive cancer patients (*P*<0.05).

**Table 3:** The prognostic significance of *Becn1* mRNA in gastric cancer by Kaplan–Meier plotter.

Clinicopathological features	Overall survival		Progression-free survival	
	Hazard ratio	*P* value	Hazard ratio	*P* value
Sex				
Female	0.62(0.42–0.9)	0.012	0.72(0.47–1.09)	0.12
Male	0.61(0.49–0.75)	5.1e-06	0.61(0.48–0.77)	3.7e-05
T				
2	0.59(0.37–0.94)	0.025	0.59(0.38–0.92)	0.019
3	0.7(0.49–0.98)	0.039	0.87(0.62–1.21)	0.4
4	0.49(0.21–1.15)	0.094	0.43(0.2–0.93)	0.029
N				
0	1.73(0.73–4.1)	0.21	1.77(0.75–4.17)	0.19
1–3	0.53(0.4–0.69)	2e-06	0.6(0.46–0.78)	8.1e-05
1	0.35(0.22–0.55)	2.3e-06	0.38(0.25–0.59)	6.1e-06
2	0.52(0.33–0.82)	0.0047	0.66(0.42–1.03)	0.066
3	0.56(0.32–0.98)	0.039	0.61(0.36–1.06)	0.077
TNM staging				
I	0.33(0.12–0.88)	0.02	2.43(0.78–7.62)	0.12
II	0.46(0.24–0.86)	0.013	0.49(0.26–0.93)	0.025
III	0.57(0.42–0.76)	0.00011	0.61(0.41–0.91)	0.015
IV	0.68(0.46–1)	0.048	0.67(0.46–0.98)	0.04
Lauren’s classification				
Intestinal-type	0.56(0.4–0.77)	0.00039	0.69(0.48–0.98)	0.04
Diffuse-type	0.55(0.38–0.79)	0.0011	0.57(0.4–0.83)	0.003
Her-2 positivity				
−	0.65(0.52–0.81)	0.00016	0.65(0.5–0.84)	0.00088
+	0.77(0.59–1)	0.051	0.7(0.5–0.99)	0.043
Treatment				
Surgery alone	0.66(0.48–0.91)	0.01	0.72(0.53–0.97)	0.03
5-FU-based adjuvant	1.68(1.18–2.39)	0.0034	1.66(1.17–2.35)	0.0044

### Becn1-Related Signal Pathways in Gastric Cancer

As summarized in **Table 4**, we conducted a GSEA to analyze *Becn1*-related signal pathways in gastric cancer. The significant enriched pathways included prostate, lung, renal, colorectal, endometrial, thyroid cancers, glioma, and leukemia, the metabolism of amino acid, lipid and sugar, and some signal pathways of insulin, MAPK, TRL, VEGF, JAK-STAT, chemokine and p53, lysosome, peroxidome and ubiquitin-mediated protein degradation (*P*<0.05).

**Table 4:** *Becn1*-enriched signal pathway in gastric cancer according to the KEGG analysis.

Name	Size	*P* value
Phosphatidylinositol signaling system	76	<0.001
Inositol phosphate metabolism	54	<0.001
Endocytosis	178	<0.001
Amyotrophic lateral sclerosis	52	<0.001
Insulin signaling pathway	136	0.001
Lysosome	119	0.001
Endometrial cancer	52	0.001
Adipocytokine signaling pathway	67	0.001
Apoptosis	85	0.001
Fatty acid metabolism	42	0.001
Sphingolipid metabolism	36	0.001
Acute myeloid leukemia	57	0.001
mTOR signaling pathway	51	0.001
Prostate cancer	88	0.001
Pathways in cancer	324	0.001
ERBB signaling pathway	87	0.001
Chronic myeloid leukemia	73	0.001
Small cell lung cancer	84	0.002
Peroxisome	78	0.005
FC gamma r mediated phagocytosis	93	0.005
Neurotrophin signaling pathway	126	0.005
Non-small cell lung cancer	54	0.005
Vasopressin regulated water reabsorption	44	0.005
Colorectal cancer	62	0.005
Ubiquitin mediated proteolysis	134	0.005
Amino sugar and nucleotide sugar metabolism	44	0.005
MAPK signaling pathway	264	0.005
Vibrio cholerae infection	53	0.005
Fructose and mannose metabolism	34	0.007
Glycolysis gluconeogenesis	59	0.008
N-glycan biosynthesis	46	0.008
Snare interactions in vesicular transport	38	0.009
Renal cell carcinoma	70	0.009
Focal adhesion	199	0.01
Toll-like receptor signaling pathway	89	0.013
Regulation of actin cytoskeleton	210	0.013
Chemokine signaling pathway	185	0.014
Lysine degradation	41	0.014
Long term potentiation	69	0.014
Pancreatic cancer	70	0.014
Adherens junction	73	0.014
Wnt signaling pathway	149	0.014
Glycosaminoglycan degradation	21	0.014
VEGF signaling pathway	75	0.014
Valine leucine and isoleucine degradation	44	0.014
Epithelial cell signaling in helicobacter pylori infection	67	0.014
JAK-STAT signaling pathway	136	0.017
NOD-like receptor signaling pathway	61	0.019
Glioma	65	0.02
Galactose metabolism	25	0.02
Thyroid cancer	29	0.02
Axon guidance	128	0.022
FC epsilon RI signaling pathway	78	0.023
Selenoaminoacid metabolism	26	0.03
Betaalanine metabolism	22	0.032
GNRH signaling pathway	100	0.032
Glycerophospholipid metabolism	71	0.032
Propanoate metabolism	32	0.032
Aldosterone regulated sodium reabsorption	41	0.032
Progesterone mediated oocyte maturation	83	0.033
Glycerolipid metabolism	42	0.033
Tight junction	129	0.034
P53 signaling pathway	66	0.034
RIG I like receptor signaling pathway	56	0.034
O-glycan biosynthesis	27	0.037
Leukocyte transendothelial migration	115	0.039
Butanoate metabolism	33	0.043
Oocyte meiosis	109	0.046

## Discussion

Beclin 1 expression was reported to cause apoptosis *via* Bax activation and Bcl-2 suppression in HeLa cells, inhibited proliferation and increased the chemosensitivity to Taxol (38). In lung cancer cells, Beclin 1 induced apoptosis and weakened invasion by up-regulating ECRG4 (39). In breast cancer cells, Beclin 1 overexpression improved cellular autophagy, inhibited cell proliferation, decreased cell apoptosis, mediated G_1_ arrest and promoted epithelial-mesenchymal transition (EMT) *via* Wnt/β- catenin pathway (40), but *Becn1* knockdown induced EMT by *via* posttranscriptional up-regulation and stabilization of Zeb1 mRNA (41). RelA-mediated *Becn1* expression was essential for ROS-induced autophagy in oral cancer cells irradiated by laser (42). Hasan et al. (43) found that HSP90 inhibitor (Gedunin) suppressed interaction between Hsp90:Beclin-1:Bcl-2, finally to inhibit autophagosome formation (Beclin-1, Atg5-12 complex, and LC3). Hu et al. (44) observed that Becn1 knockdown markedly promoted motility and invasion of colorectal cancer cells with STAT3 phosphorylation *via* the interaction between STAT and JAK2. Cheng et al. (45) found that Beclin 1 increased the migration of non-small cell lung cancer cells *via* interaction with Vimentin, followed by its K48-linked ubiquitination *via* ubiquitin-specific peptidase 14. Here, Beclin 1 was demonstrated to suppress proliferation, migration, invasion and tumor growth, and induce cell cycle arrest, apoptosis, autophagy and chemoresistance in gastric cancer cells, whereas versa for *Becn1*-silencing cells, suggesting that Beclin 1 can be used as a gene therapy target of gastric cancer if its chemoresistant induction could be avoided or alleviated. Additionally, gastric lesions were not observed from monoallelic conditional KO mice of *Becn1* in pit, parietal and chief cells respectively although spontaneous lymphomas, liver and lung cancers were detected in *Becn1*-/+ mice (23). Reportedly, microadenoma, macroadenoma to invasive well-differentiated adenocarcinoma were rapidly observed in the gastric Lgr5+ stem cells with the double deletion of Smad4 and PTEN using Lgr5-Cre mice (46). Our findings support the opinion that Becn-1-mediated gastric cancer might originate from local stem cells with genetic alteration, but not the differentiated cells. Additionally, *Becn1*-induced gastric carcinogenesis possibly needs the exposure to chemical carcinogen.

Bax can open the mitochondrial voltage-dependent anion channel during apoptosis, which is inhibited by interaction with Bcl-2 on the mitochondrial membrane (47). Consequently, Bax overexpression and Bcl-2 underexpression in gastric cancer cells may account for the inductive effect of Beclin 1 on apoptosis *via* mitochondrial pathway. Reportedly, activated p38 MAPK phosphorylates MAPKAP kinase 2 to phosphorylate the transcription factors (e.g. ATF2, Mac, and MEF2) and subsequently to mediate cell survival. PI3K/Akt activation results in drug resistance and cell proliferation. We observed p-p38 overexpression in *Becn1*-ovexpressing or silencing gastric cancer cells, but both p-PI3K and p-Akt hyperexpression was seen in *Becn1*-ovexpressing, but not *Becn1*-knockdown cells, suggesting that PI3K/Akt signal pathway might be involved in the effects of Beclin 1, but not p38. According to the literature, MDR1 and LRP1 are mainly responsible for drug resistance due to their ATP-dependent efflux pumping (47). ING5 overexpression also caused chemoresistance in neuroblastoma, glioma, gastric cancer, lung cancer, ovarian cancer and breast cancer cells (48–52). Cellular stress or increased metabolic demand activates autophagy, which can cause therapeutic resistance (47). Our results hinted that the Beclin 1-mediated chemoresistance might be due to autophagy and the hyperexpression of MDR1, LRP1, and ING5. *Becn1* overexpression was found to markedly attenuate the ability of gastric cancer cells to migrate and invade possibly due to VEGF hypoexpression because VEGF may promote mobility and proliferation of gastric cancer cells. In the study, E-cadherin hyperexpression, and Twist1 and Zeb2 hypoexpression in gastric Becn1 transfectants were closely linked to MET because Zeb2 and twist promote EMT process with E-cadherin underexpression. Therefore, the inhibitory of Beclin 1 on migration and invasion was dependent on MET in gastric cancer cells.

TGF-β1-induced autophagy linked β-catenin and Smad signaling to promote EMT of mouse kidney proximal tubular epithelial C1.1 (SV40 transformed) cells by disrupting E-cadherin/β-catenin- mediated cell-cell contact *via* ILK overexpression (53). SIRT1, SIRT6 and SPHK1 induced EMT by up-regulating autophagy-linked lysosomal degradation of E-cadherin in melanoma and hepatocellular cells *via* Beclin 1-E-cadherin cascade respectively (54–56). FSTL1 was demonstrated to induce EMT and airway remodeling by activating autophagy in asthma (57). HMGB1 also induced apoptosis and EMT in association with autophagy through the upregulated expression of DDR1 and the downregulation of the phosphorylation of mTOR following H/R injury in H9c2 cells (58). Persistent hypoxia induced autophage disorders, which could cause down-regulated E-cadherin and down-regulated MMP-9, thus promoting invasiveness of placenta trophoblasts (59). GRIM-19 suppressed hypoxia-triggered invasion and EMT by inhibiting hypoxia-induced autophagy through inactivation HIF-1α/STAT3 signaling axis (60). HIF-1α-mediated autophagy promoted EMT and metastatic ability of CD133+ pancreatic cancer stem-like cells during intermittent hypoxia (61). Endoplasmic reticulum stress induced EMT through autophagy *via* c-src kinase activation (62). In contrast, Catalano et al. (63) reported that autophagy induction impaired migration and invasion by reversing EMT in glioblastoma cells, in line with our inductive effects of Beclin 1 overexpression on E-cadherin expression in gastric cancer. In combination of these findings, we speculated that the regulatory correlation between Beclin 1 and E-cadherin depended on the cell types.

Reportedly, loss and down-regulation of Beclin 1 expression was immunohistochemically observed in esophageal adenocarcinoma (64), colorectal cancer (65), breast cancer (66), but versa in pancreatic cancer (67). Here, we performed meta-analysis and found that down-regulated Beclin 1 expression in gastric cancer was positively linked to lymph node metastasis, TNM staging, dedifferentiation and poor prognosis, in agreement with our bioinformatics findings. Reportedly, loss of Beclin-1 in cancer cells and Beclin 1 overexpression in stromal mesenchymal cells were closely linked to local recurrence and lymph node metastasis in breast cancer (68). Beclin-1 expression was related to HBV infection status and the grade of hepatocellular carcinoma (HCC) (69). In the hypoxic group, it was negatively correlated with high tumor grade, advanced stage, large size, and multifocal tumors of HCC, while positively with TNM stage and liver metastasis of gallbladder carcinoma (70), and with worse recurrence in intrahepatic cholangiocarcinoma (71). Beclin 1 expression was reported negatively correlate with tumor grade, lymph node involvement, TNM stage, tumor size, dedifferentiation, and recurrence of lung cancer (72). These findings indicated that its down-regulation contributed to gastric carcinogenesis and progression as a molecular marker.

*Becn1* expression loss was demonstrated to act as a negative prognosticator in ovarian cancer patients receiving platinum-based chemotherapy (73). Tang et al. (74) found that low *Becn1* expression was associated with poor prognosis in breast cancer as an independent predictor, but the converse was true for breast cancer patients receiving tamoxifen treatment (75). Beclin 1 was considered as a dependent factor for favorable prognosis of the colorectal cancer patients (65). It was the same for ovarian clear cell carcinomas (76) and multiple myeloma (77). In contrast, Beclin 1 was significantly correlated with short disease-free survival and overall survival of pancreatic ductal adenocarcinoma (67). Here, meta- or bioinformatics analysis showed that either Beclin 1 protein or mRNA expression was positively linked to the favorable prognosis of the patients with gastric cancer. Taken together, Beclin 1 might be considered as a potential marker for the prognosis of the gastric cancer patients at either mRNA or protein level.

Down-regulated *Becn 1* expression in gastric cancer was positively correlated with aggressiveness and worse prognosis at either mRNA or protein level as a molecular marker. *Becn1* KO in pit, parietal or chief cells couldn’t induce gastric carcinogenesis. It might be employed as a potential target for gene therapy of gastric cancer patients if its chemoresistant induction would be avoided and alleviated.

## Data Availability Statement

The raw data supporting the conclusions of this article will be made available by the authors, without undue reservation.

## Ethics Statement

The animal study was reviewed and approved by The Affiliated Hospital of Chengde Medical University.

## Author Contributions

H-cZ, SZ, HX, E-hZ, H-mJ, and C-lH designed and carried out the experiments, and HZ wrote the draft. All authors read and approved the final manuscript. All authors contributed to the article and approved the submitted version.

## Funding

This study was supported by Award for Liaoning Distinguished Professor, and National Natural Scientific Foundation of China (81672700).

## Conflict of Interest

The authors declare that the research was conducted in the absence of any commercial or financial relationships that could be construed as a potential conflict of interest.
